# Subclinical Mastitis in Dairy Animals: Incidence, Economics, and Predisposing Factors 

**DOI:** 10.1155/2014/523984

**Published:** 2014-06-30

**Authors:** Mukesh Kr. Sinha, N. N. Thombare, Biswajit Mondal

**Affiliations:** ^1^Directorate of Water Management, Bhubaneswar, Odisha 751023, India; ^2^Indian Veterinary Research Institute, Izatnagar 243122, India; ^3^Central Rice Research Institute, Cuttack, Odisha 753006, India

## Abstract

A study was conducted to assess the incidence and economics of subclinical form of bovine mastitis in Central Region of India. Daily milk records of 187 animals during three seasons were collected and subjected to analysis. The economic loss due to reduction in yield, clinical expenses, and additional resources used were quantified and aggregated. The losses due to mastitis in monetary terms were estimated to be INR1390 per lactation, among which around 49% was owing to loss of value from milk and 37% on account of veterinary expenses. Higher losses were observed in crossbred cows due to their high production potential that was affected during mastitis period. The cost of treating an animal was estimated to be INR509 which includes cost of medicine (31.10%) and services (5.47%). Inadequate sanitation, hygiene, and veterinary services were the main predisposing factors for incidence and spread of mastitis as perceived by the respondents.

## 1. Introduction

Among the animal diseases which affect the profitability of rearing animals, mastitis is considered to be one of the expensive diseases in terms of production losses [1, 2]. The losses are the potential revenues not earned, while the control costs are actual expenditures related to treatments, preventive measures, and additional labour used by them [3]. The economic calculations of production losses and knowledge of the cost component are very essential in farmer's decision to develop control mechanism. Many studies have been conducted on preventive and microbial aspects of this disease as well as simulative form and few studies are based on data of the field farms to estimate production-related losses and treatment costs [4–7]. Predisposing factors for mastitis incidence highly depend upon type of breed, stage of lactation, management practices, and awareness of the dairy farmers. Scant literature is available on quantification of region-specific economic effects of subclinical form of mastitis where visible abnormalities such as udder swelling, hardness of the affected quarter, pain, and watery milk remain absent. The present study analyzed the incidences and economic losses from such kind of infections which generally remain unnoticed but rendered to huge loss in quantity as well as quality of milk yield. Quantification of such losses from the disease would help to take preventive measures for avoiding such losses and aid decision making in livestock health management among the small dairy farmers in the region.

## 2. Materials and Methods

To gather data on incidence rate and economic losses due to mastitis, two blocks, namely, Baramati and Daund, from Pune district located in Central region of India were selected based on substantial population of crossbred cows. About 28 farms were selected randomly from a cluster of villages and 187 animals were presumed by them as mastitis affected (subclinical forms without any visible abnormalities in udder or flakes, clots, and watery appearance in milk). Information on season-wise number of different categories of animals affected was collected and incidence rate was calculated as the number of affected cases occurring in the specified population during a specified time period divided by the average number of individuals in that population during that specified time period. It is also a way of measuring the risk that a susceptible individual in a population has chances of contracting a disease during a specified time period.

To account for the variation in milk yield, minimum 15 days' milk yields before and after the treatment were recorded. For calculating the losses due to the disease, information on average daily milk yield, price of milk per liter, reduction in milk yield during the affected period, number of days of illness, discarded milk during affected periods, veterinary expenses, labour charges, stall hygiene, and milk hygiene was collected from the sample farmers. The loss of milk during treatment period was calculated by the difference between average milk potential of each animal before and after treatment and it was multiplied by prevailing milk price to arrive at value of milk loss due to the disease. For quantification and aggregation of economic losses due to incidence of the disease owing to reduction in milk yield, additional resources used, and expenditures made for treatment/prevention the following equation [8] was used:

(1) $$\begin{array}{c} \text{Loss}\;\;\text{per}\;\;\text{animal}=\left\{\left(M_{i}P_{m}+C_{t}\right)D+\left(V-V_{d}\right)\right\}, \end{array}$$

where *D* is average duration of the infection; *M*_*i*_ is milk loss per animal per day; *P*_*m*_ is price of milk per litre; *C*_*t*_ is cost of treatment and prevention per animal per day; *V* is market value of adult animal; and *V*_*d*_ is market value of affected animal.

A focus group discussion was held to identify the major predisposing factors which might influence the spread of this disease among herds. Further, the respondents were asked to rank the factors in terms of severity and frequency of occurrence of the disease. Finally, the ranks were converted to scores and final rank was calculated using Garrett's ranking technique [9] with the following formula:

(2) $$\begin{array}{c} G_{i}=\frac{100\left(R_{ij}-0.50\right)}{N_{j}}, \end{array}$$

where *G*_*i*_ is percentage position of *i*th factor, *R*_*ij*_ is rank given for the *i*th factor by the *j*th respondent, and *N*_*j*_ is the number of factors ranked by the *j*th respondent.

## 3. Result and Discussion

### 3.1. Social Attribute of Farm Owners

The socioeconomic characteristics of the respondent are very much linked to their ability to take decision, physical wellbeing, work efficiency, and level to tackle the adversaries at farm. The farmer status and management practices have been recorded at the time of investigation and presented in Table 1. The majority (71.83%) of respondents belong to middle age groups (30–50 years) and 68% of them attended schools only up to primary standards. About 39.29% of the respondents habited in* Katcha* house and 7.14% farm owners were unable to maintain even a shed for their animals. Grazing was not commonly practiced in the area and 90% animals were of stall-fed category.

### 3.2. Incidence of the Disease

The incidence of mastitis distributed over season, stage of lactation, and type of animal are presented in Table 2. The results indicated that incidence of reported cases was the highest during monsoon season and the lowest during summer. Around 46% of affected animals were observed to lie within 30–90-day period of lactation. Species-wise highest incidence of the disease was observed in crossbred cows which implied that crossbred cows are more susceptible to the disease. Less incidence of the disease in buffaloes might be due to the thick and compact epithelium, thick keratin layer, and thick muscle sphincter in streak canal of udder of buffaloes as compared to crossbred cows and the similar results obtained earlier by Saini et al. [10].

### 3.3. Economic Losses due to Mastitis in Dairy Animals

Table 3 presents the component wise economic losses due to incidence of the disease. The overall loss from dairy animals was estimated at INR1390 per lactation, in which major losses were owing to the reduction in return from milk yield to the extent of 48.53% followed by veterinary expenses which accounts for 36.57% of the total loss. In crossbred cows, loss due reduction in yield was observed to be INR700, while in buffaloes it was accounted to be INR364. A higher loss was estimated in crossbred due to its high production potential which is otherwise lost during mastitis period. The veterinary expenses in crossbred cow were also observed more (INR 582) due to its high cost of rearing and sophisticated management practices. Additional labour charges along with the cost of sanitation and hygiene were the other components considered to calculate the total economic loss.

Besides reduction in milk yield, various antibiotics, analgesics, anti-inflammatory drugs, and intramammary infusions were used for treating affected animals which definitely have reduced their market value. Kumar et al. [11] reported that a complete fibrosis of one quarter leads to an average decrease in market value by INR 4000 and INR2500 for crossbred cows and buffaloes, respectively. Accordingly, based on population statistics as per 18th livestock census (2007) and trend of mastitis incidence observed in this study, the economic losses per annum were estimated to be INR 9.09 million and INR85.23 million for Pune district and Maharashtra state, respectively.

### 3.4. Identification and Ranking of Predisposing Factors for Spread of Mastitis

Focus group discussion among the villagers revealed a variety of factors related to housing, feeding, milking management, and sanitation as well as clinical facilities available which might influence the spread of the disease. The factors were clubbed and a consolidated list was prepared and the respondents were asked to rank the factors in terms of severity and frequency of occurrence of the disease and a final rank was prepared using Garrett's ranking technique (Table 4).

The results revealed that about 84% of the opinions from farmer's responses was for poor sanitation and hygiene as the major factors which influence the spread of the disease, while 80% opinions was for nonavailability of veterinary services followed by poor literacy and awareness which scored 74% of opinions. Sociocultural practices and agroclimatic condition, though identified, were not perceived major factors for spreading of the disease.

## 4. Conclusions

The economic consequences of losses due to subclinical form of mastitis were assessed in terms of reduction in milk yield, medicine and veterinary expenses incurred, and additional resources used. The overall losses were estimated at INR1390 per lactation, in which around 49% was due to reduction of milk production alone followed by veterinary expenses which accounted for 37% of the total loss. In crossbred cows and buffaloes, losses due to reduction in milk yield were worked out at INR700 and INR364, respectively. A greater loss in crossbred cows was observed due to their high production potential which was affected during the infection period. However, it is very difficult to generalize and compare the losses across the farms due to specific nature and management practices as well as farm-specific economic characteristics. Inadequate sanitation, poor hygiene, and lack of proper veterinary services were the major predisposing factors for occurrence and spread of the disease. An effective extension service is needed to create awareness about the requirements of proper hygiene and adoption of other preventive measures by the rural farmers to reduce the losses due to mastitis incidence.

## Figures and Tables

**Table 1:** Sociopersonal attributes of sample dairy farm owner.

Age group	%	Housing type	%	Education level	%	Management of dairy animals	%
Young (<30)	10.71	*Katcha *	39.29	Illiterate	7.14	Grazing	3.57
Middle (30–50)	71.43	*Pucca *	53.57	Primary	67.86	Stall-fed	89.29
Old (>50)	17.86	No shed	7.14	High school and above	25.00	Mixed	7.14

**Table 2:** Number of mastitis incidences over season, stage of lactation, and species of animals.

Season	Affected animals	Stage of lactation	Affected animals	Species	Affected animals
Monsoon (June–September)	92 (4.68)	<30 days	63 (3.20)	Crossbred cows	172 (9.88)
Winter (October–January)	52 (2.64)	30–90 days	86 (4.37)	Indigenous cow∗	—
Summer (February–May)	43 (2.19)	>90 days	38 (1.93)	Buffaloes	15 (6.66)

*No indigenous cow was reported in the sample having mastitis.

Figures in parenthesis indicate rate of incidence with respect to total population of dairy animals with the sample farms.

**Table 3:** Components of losses (in INR) due to mastitis in farm animals.

Type of losses	Crossbred cows	Buffaloes	Overall
(A) Production loss	700.18 (43.95)	363.75 (40.75)	674.74 (48.53)
(i) Milk yield loss	503.04 (31.58)	273.75 (30.67)	485.69 (34.93)
(ii) Loss from discarded milk	197.14 (12.37)	90.00 (10.08)	189.05 (13.60)
(B) Veterinary expenses	582.15 (36.54)	356.67 (39.97)	508.52 (36.57)
(i) Medicine	505.36 (31.72)	290.00 (32.50)	432.50 (31.10)
(ii) Services	76.79 (4.82)	66.67 (7.47)	76.02 (05.47)
(C) Sanitation	66.07 (3.45)	53.67 (6.01)	65.09 (4.68)
(i) Stall hygiene	47.68 (2.30)	37.50 (4.20)	46.87 (03.37)
(ii) Milk hygiene	18.39 (1.15)	16.17 (1.81)	18.22 (01.31)
(D) Miscellaneous	144.47 (9.06)	118.33 (13.26)	142.11 (10.22)
(i) Additional labour	98.93 (6.21)	78.33 (8.78)	96.99 (6.98)
(ii) Equipment and so forth	45.54 (2.85)	40.00 (4.48)	45.12 (3.24)

Total	1592.87 (100.00)	892.42 (100.00)	1390.46 (100.00)

Figures in parentheses indicate percentage of total loss.

**Table 4:** Predisposing factors for spread of mastitis in farm animals.

Factors	Percent position	Garrett score	Final rank
Agroclimatic condition	19.17	68.00	V
Sociocultural practices	16.67	69.00	IV
Poor literacy and awareness	11.50	74.00	III
Poor sanitation and hygiene	4.33	84.00	I
Nonavailability of veterinary services	6.67	80.00	II
